# Strain in Silica-Supported Ga(III) Sites: Neither Too Much nor Too Little for Propane Dehydrogenation Catalytic Activity

**DOI:** 10.1021/acs.inorgchem.0c03135

**Published:** 2021-02-05

**Authors:** C. S. Praveen, A. P. Borosy, C. Copéret, A. Comas-Vives

**Affiliations:** ‡ Department of Chemistry and Applied Biosciences, ETH Zürich, Vladimir Prelog-Weg 1−5, CH-8093 Zürich, Switzerland; § Department of Chemistry, Universitat Autònoma de Barcelona, 08193 Cerdanyola del Vallès, Catalonia, Spain

## Abstract

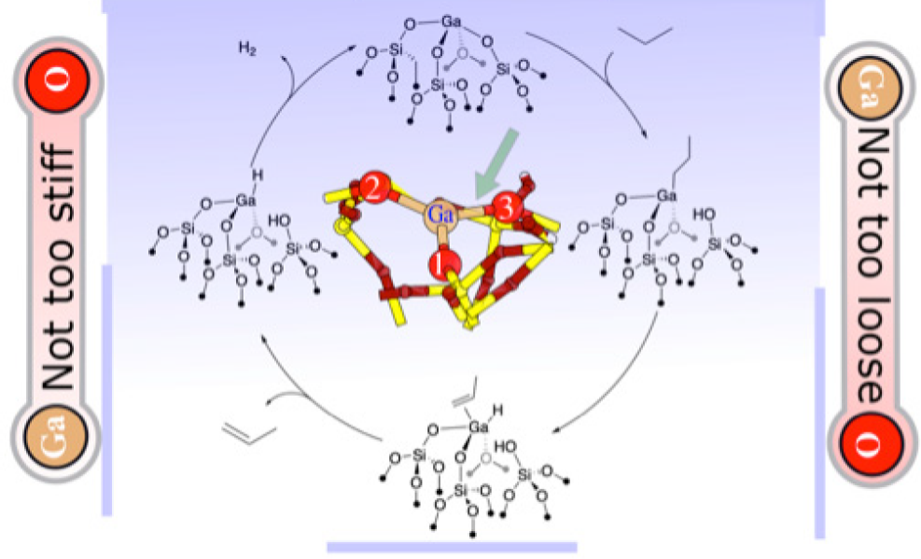

Well-defined Ga(III) sites on SiO_2_ are highly active, selective, and stable catalysts in the propane dehydrogenation (PDH) reaction. In this contribution, we evaluate the catalytic activity toward PDH of tricoordinated and tetracoordinated Ga(III) sites on SiO_2_ by means of first-principles calculations using realistic amorphous periodic SiO_2_ models. We evaluated the three reaction steps in PDH, namely, the C–H activation of propane to form propyl, the β-hydride (β-H) transfer to form propene and a gallium hydride, and the H–H coupling to release H_2_, regenerating the initial Ga–O bond and closing the catalytic cycle. Our work shows how Brønsted–Evans–Polanyi relationships are followed to a certain extent for these three reaction steps on Ga(III) sites on SiO_2_ and highlights the role of the strain of the reactive Ga–O pairs on such sites of realistic amorphous SiO_2_ models. It also shows how transition-state scaling holds very well for the β-H transfer step. While highly strained sites are very reactive sites for the initial C–H activation, they are more difficult to regenerate. The corresponding less strained sites are not reactive enough, pointing to the need for the right balance in strain to be an effective site for PDH. Overall, our work provides an understanding of the intrinsic activity of acidic Ga single sites toward the PDH reaction and paves the way toward the design and prediction of better single-site catalysts on SiO_2_ for the PDH reaction.

## Abstract 2

We performed computational calculations of Ga(III) single sites on realistic amorphous models of SiO_2_ to evaluate their catalytic activity toward the propane dehydrogenation reaction. Our results show that a balance in strain is key, in which neither too stiff nor too loose Ga−O bonding is needed to obtain the highest catalytic activity.

## 1 Introduction

The high demand of light olefins^1^ and the large abundance of shale gas,^2^ mostly constituted of light alkanes, have stimulated interest in on-site propane dehydrogenation (PDH).^1,3^ PDH involves activation of the C(sp^3^)–H bond of propane as a first step, which is still nowadays a very challenging reaction.^4^ Because of the highly endothermic nature of alkane dehydrogenation, this reaction is generally carried out at 550 °C to obtain reasonable conversion to the alkene product. The two historical heterogeneous catalysts that are used for this reaction in industry correspond to alumina-supported PtSn nanoparticles and the CrO_*x*_/Al_2_O_3_ system, also known as the Houdry or Catofin catalysts.^5^ Recent research developments have also helped to launch a PtGa-based catalyst.^6^ The Cr-based catalyst is thought to have Cr(III) active sites dispersed on alumina. Among alternative supported catalysts, Ga-based materials are particularly noteworthy. For instance, Ga-exchanged zeolites can convert light alkanes such as propane directly into aromatics and H_2_ in a process proposed to involve a tandem dehydrogenation–aromatization process.^7−13^ Ga_2_O_3_ also promotes PDH reaction, but it suffers from fast deactivation, presumably because of reduction of the catalyst under reaction conditions.^14,15^ More recently, silica-supported well-defined Ga(III) single-site catalysts have been developed^16^ using a combined approach of surface organometallic chemistry^17−23^ and a thermolytic precursor using [Ga(OSi(O*t*Bu)_3_)_3_(THF)] as a molecular precursor.^16^ This approach generates tetracoordinated Ga sites, [(≡SiO)_3_Ga(XOSi≡)] (X = −H or ≡Si), according to IR, X-ray absorption near-edge structure, and extended X-ray absorption fine structure (EXAFS) analyses. This catalyst displays high activity and selectivity towards propene as well as remarkable stability compared to Ga_2_O_3_ and other single-site catalysts based on Fe, Co, and Zn.^24−28^ PDH is proposed to involve three main elementary steps: the C–H activation of propane on the Ga–O pair site, formation of a Ga–propyl intermediate and an OH group, the subsequent β-hydride elimination, forming a hydride and propene, and the decoordination of propene and H–H coupling to regenerate the initial Ga–O pair. Previous works by Sauer et al. on ethane dehydrogenation also considered the same sequence of reactions on ethane dehydrogenation catalyzed by Cr(III) sites.^29^ Other reaction steps, such as regeneration of the propyl group via σ-bond metathesis of an incoming propane molecule releasing H_2_ and regenerating propyl, are significantly more energy-demanding, as we showed recently for our work on the Cr(III) system.^30^ Thus, the latter step was not considered in the present study.

To evaluate the catalytic activity of well-defined silica-supported single-site catalysts by first principles, cluster models have typically been used because of the simplicity of these models and their associated low computational cost, but such models do not account for the expected site heterogeneity on an amorphous support like SiO_2_ and cannot be used to model highly strained sites that are generated upon thermal treatment at high temperature.^31^ In fact, the high degree of heterogeneity has been evidenced on so-called silica-supported single-site catalysts by luminescence spectroscopy^32^ and magnetic properties^33^ as well as the polydispersity of polyethylene obtained on the corresponding Cr(III) systems.^30,34,35^ We have also recently shown that the use of an amorphous SiO_2_ model^36^ can account for strain in the Cr(III)/SiO_2_ catalyst and allows for an explanation of the reactivity of this catalytic system toward olefin polymerization.^37,38^ In our previous study for the Cr(III)/SiO_2_ system, we have shown that there is a large variability of the reactivity of the Cr–O pairs, and those that are more strained are significantly more reactive than the ones that are less elongated and therefore less strained and prone to react either by cleaving the C–H bond or by inserting the ethylene into the Cr–O bond, forming an oxachromacycle.^37,38^

Here, we evaluate the reactivity of isolated Ga(III) sites with different degrees of strain on amorphous silica toward PDH reaction using first-principles calculations. We show the high variability of the reactivity from site to site and encounter Brønsted–Evans–Polanyi (BEP) relationships for the three main reaction steps in the PDH reactions, serving as a guide for future screening studies. We also propose that the most efficient sites toward the PDH reactions in the Ga(III)/SiO_2_ system display the “right” balance of strain, where the sites with intermediate strain and not the most strained ones are the most efficient for PDH because they both can activate the C–H bond of propane effectively and are easier to regenerate than the most strained sites, thus yielding an overall more efficient catalysis.

## 2 Results

### 2.1 Construction of Ga(III) Sites

Ga(III)/SiO_2_ models are constructed using a recently developed amorphous silica model,^36^ which corresponds to a slab of dimensions 21.4 Å × 21.4 Å × 34.2 Å and contains 372 atoms. The silica model exposes five isolated silanol (SiOH) groups and has a surface SiOH density (1.1 OH nm^–2^) close to the density found for silica partially dehydroxylated under vacuum at 700 °C (SiO_2-700_, 0.8 OH nm^–2^), which is used experimentally to prepare well-defined Ga(III) sites and related systems.^16^

The amorphous SiO_2_ model is obtained from a fully hydroxylated amorphous silica model by direct condensation of adjacent SiOH groups and by surface reconstruction steps involving SiO_2_ migrations. The amorphous silica model has a high degree of heterogeneity, as evidenced by the large variability in the energetics associated with the dehydroxylation steps. The average Si–O distances of the siloxane bridges formed upon surface dehydroxylation serve as descriptors of the strain present on the silica surface.^36^ The Ga sites are introduced to the silica model by substituting surface “SiOH” groups by Ga^3+^, i.e., turning (≡SiO)_3_SiOH sites into (≡SiO)_3_Ga sites, as previously carried out to build the corresponding Cr(III) sites.^37,38^ This model provides five types of Ga(III)/SiO_2_ models, models I–V. In contrast to Cr, we also consider the coordination of an additional siloxane group to Ga because EXAFS data pointed to the presence of this additional siloxane bridge.^16^ Among the various Ga(III) sites, site I has one siloxane group in close contact with the Ga center, with a distance equal to 2.465 Å, and can be considered as a model for [(≡SiO)_3_Ga(≡SiOSi≡)] sites. Other similar sites are built by placing a siloxane bridge at 2.3 Å for all of the II, III, IV and V sites. Geometry optimization yields the final structures. Of them, site III yields another model for [(≡SiO)_3_Ga(≡SiOSi≡)], with a Ga···O(Si≡) bond of 2.479 Å, referred to as the site mod-III, which is 2.4 kcal·mol^–1^ less stable than the initial site III (which does not contain a siloxane bound to Ga). For the latter two structures, the Ga···O(Si≡) bond is rather long, ranging from 2.465 to 2.479 Å, possibly because of the high constraint imposed by siloxane groups near the Ga single site. Therefore, in order to consider a less constrained and shorter Ga···O bond, which might also be present on the real system, we added a siloxane ligand to the Ga–O III-site, modeled by H_3_Si–O–SiH_3_ to evaluate the effect on the reactivity on this tetracoordinated Ga site, with a shorter bond between Ga and siloxane. For the latter system, the additional Ga–O bond is 2.036 Å long, and the group has a binding energy to the Ga center equal to −18.4 kcal·mol^–1^, with respect to the free ligand in the gas phase.

Site III is the most stable one among those that we constructed. The relative stabilities of the initial tricoordinated sites are given in Table S1. However, because of the construction method that we adopted (vide supra) and the fact that there are many different degrees of strain on the Si–OH groups of SiO_2_, in our opinion, this does not rule out the fact that sites “less stable” based on this construction are not formed in the real case. At the end, most Si–OH groups react with molecular Ga and generate Ga single sites after calcination.

**Figure 1:**
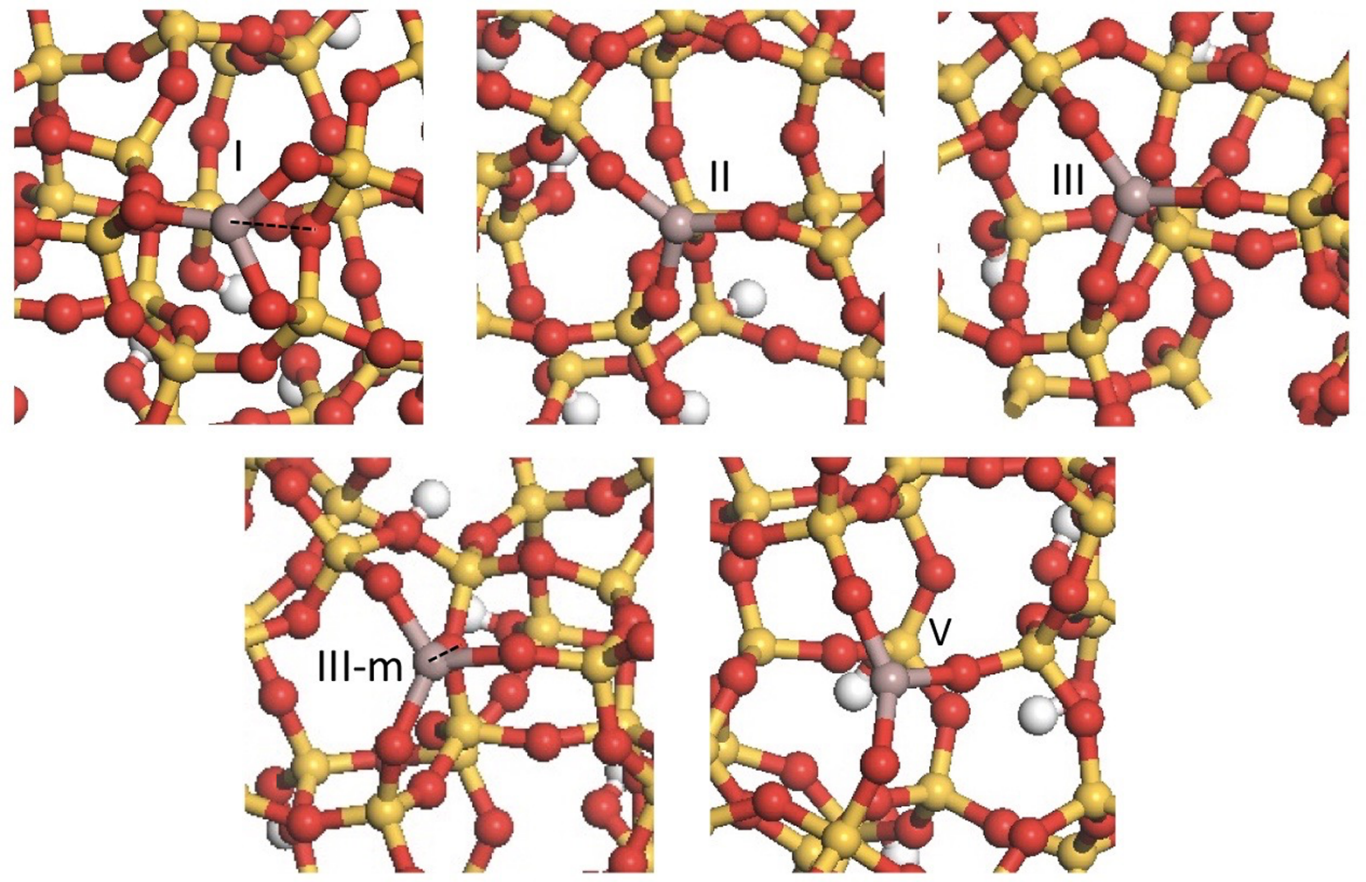
Ga sites on the amorphous model of SiO_2_ (I, II, III, III-mod, and V sites).

We also investigate the reactivity of selected tricoordinated Ga(III) sites toward propane for the II–O3, III–O2, V–O2, and V–O3 Ga–O pairs in view of their higher reactivity as found in our previous study on the related Cr(III) sites.^37^ In addition, we also investigate the tetracoordinated Ga(III)–O pairs: I–O3 and III-mod–O2. Figure 1 shows all of the constructed tricoordinated and tetracoordinated Ga sites on the amorphous SiO_2_ model. In the Supporting Information, all of the geometrical characteristics for each Ga site are given in detail (Figures S1–S6), i.e., Ga–O distances, O–Ga–O angles, and O–Ga–O–O dihedral angles.

### 2.2 Evaluation of PDH on the Selected Ga(III)/SiO_2_ Sites

**Scheme 1:**
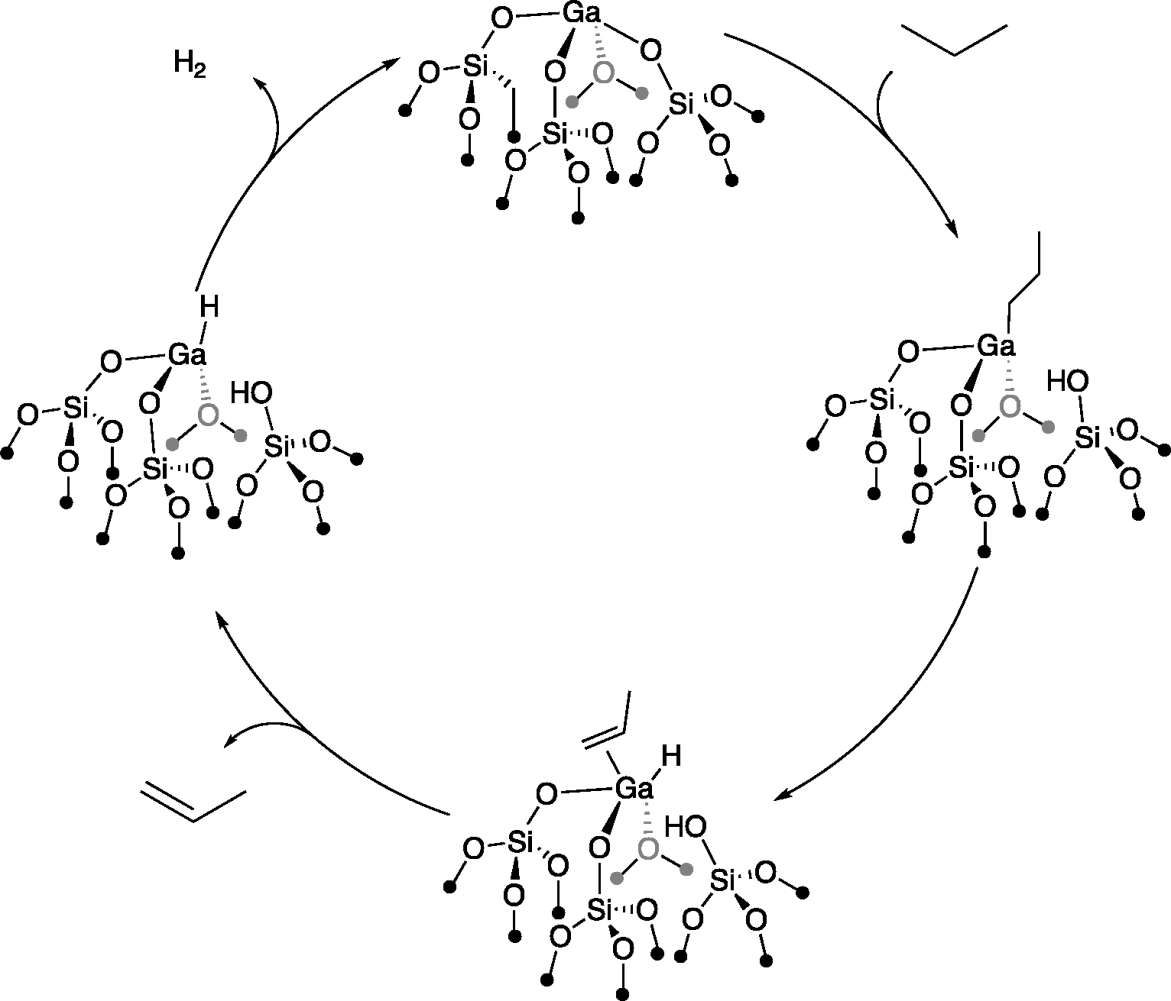
Proposed Reaction Mechanism for the Dehydrogenation of Propane on Ga(III)/SiO_2_ Sites

On these selected sites, we evaluate the PDH pathway (Scheme 1),^39^ which involves (1) C–H bond activation of propane on the Ga site, forming a Ga–propyl intermediate and an OH group (step 1), (2) subsequent β-H transfer, forming a hydride and propene (step 2), and (3) H–H coupling (step 3) following the decoordination of propene to regenerate the initial Ga sites.

### 2.3 Step 1: C–H Bond Activation of Propane

Among the evaluated tricoordinated Ga–O pairs II–O3, III–O2, V–O2, and V–O3, the Ga–O pairs involving site V are the most reactive ones, in line with the previous finding regarding the C–H activation of ethylene and propene on analogous Cr sites.^37,38^

In Table 1, we have summarized the energy barrier heights (Δ*E*^⧧^) and reaction energies (Δ*E*) for the C–H activation of propane (in kcal mol^–1^) on the evaluated Ga–O pairs. From the table, it is clear that, among all of the Ga–O pairs, the C–H activation process on V–O2 is highly exoenergetic, with a reaction energy of −65.0 kcal·mol^–1^, and it is associated with an energy barrier of 14.5 kcal·mol^–1^. The V–O3 Ga–O pair is associated with a significantly lower exoenergetic reaction energy of −25.2 kcal·mol^–1^ and a higher energy barrier of 18.5 kcal·mol^–1^. The next most reactive site for this first C–H activation step of propane is III–O2, with a thermodynamically favorable reaction energy of −23.8 kcal·mol^–1^ and an energy barrier of 21.8 kcal·mol^–1^. Finally, the C–H activation of propane on the last evaluated tricoordinated Ga–O pair, II–O3, displays a reaction energy and an energy barrier of 1.3 and 25.5 kcal·mol^–1^, respectively. We then evaluated the reactivity on tetracoordinated Ga sites, in particular on the I–O3 sites and the II-mod–O2 Ga–O sites. The tetracoordinated Ga–O pairs I–O3 and III-mod–O2 contain an additional siloxane bridge coordinating to Ga at 2.465 and 2.479 Å, respectively, and the Ga–O distance is significantly elongated in the transition state (TS) with values of 2.759 and 3.410 Å, while the distances are 3.121 and 4.183 Å in the resulting Ga–alkyl species. The C–H activation on the Ga–O I–O3 pair has an energy barrier equal to 25.1 kcal·mol^–1^ and a reaction energy exoenergetic by 22.9 kcal·mol^–1^. Therefore, it presents a relatively high energy barrier but a rather favorable reaction energy. The II-mod–O3 Ga–O pair corresponds to a modification of the II–O3 site, in which a siloxane group is closer to the Ga center by ca. 0.2 Å with respect to the II–O3 site. Nevertheless, the TS corresponding to the C–H activation is the same in both cases, which thus have the same energy. Therefore, although the respective starting minima of II–O3 and II-mod–O3 are different, both structures lead to the same TSs and also to the same products. As mentioned earlier, formation of the Ga–O siloxane bond from II–O3 to II-mod–O3 is endoenergetic by only 2.4 kcal·mol^–1^. Thus, the energy barrier and reaction energy of the C–H activation of propane are practically the same for II-mod–O3 and II–O3, being only slightly more feasible for the former than for the latter. Finally, we evaluated the Ga–O III–O2 site with the siloxane ligand, H_3_Si–O–SiH_3_, bonded to Ga. In this case, the reaction energy of the C–H activation is −23.4 kcal·mol^–1^, which is similar to the same site without the additional siloxane group bonded to Ga. Thus, overall it is likely that, if present in the Ga@SiO_2_ catalytic structure, the siloxane bridge bonded to Ga does not significantly impact the C–H bond activation step. Recently, Das et al. has reported the effect of siloxane ring strain on the formation of coordinately unsaturated metal sites on silica.^40^ They have reported that at low temperature, because of the dominance of large siloxane rings, normally tetracoordinated Ga(III) sites are stabilized. On the other hand, when silica is pretreated over 700 °C, tricoordinated Ga(III) sites are more plausible to be stabilized because of the preferred formation of small siloxane rings. The lower coordinated sites are demonstrated to be more reactive for the C–H activation, consistent with the higher activity for the tricoordinated Ga(III) sites. In addition, also tricoordinated sites of different Ga_2_O_3_-based catalysts have been proposed as their active catalytic centers.^41^ Finally, it is worth mentioning that there is a certain correlation (*R*^2^ = 0.77) between the activation energy of the TS and reaction energy, i.e., the BEP relationship when considering the following sites: I–O3, II–O3, III–O2, V–O2, and V–O3. The II-mod–O2 Ga–O pair was not considered in the evaluation of the BEP relationship. This BEP relationship is depicted as a graph in Figure S7.

**Table 1:** Energy Barrier Heights (Δ*E*^⧧^) and Reaction Energies (Δ*E*) for the C–H Activation of Propane (in kcal·mol^–1^) on the Evaluated Ga–O Pairs

Ga–O pair	Δ*E*^⧧^ (kcal·mol^–1^)	Δ*E* (kcal·mol^–1^)
I–O3	25.1	–22.9
II–O3	25.5	1.3
III–O2	21.8	–23.8
V–O2	14.5	–65.0
V–O3	18.5	–25.2

### 2.4 β-H Transfer and Propene Decoordination

Following the C–H bond activation step, which yields Ga–alkyl and O–H groups, the next step corresponds to a β-H transfer, which forms Ga–H and a propene coordinated to the Ga center. The relative energy barrier (Table 2) for this step is significantly higher than that for the C–H activation of propane, and this step is highly endoenergetic. In this case, the relative energy barriers take values ranging from 41.7 to 51.8 kcal·mol^–1^, while the reaction energies are all endothermic, with values varying between +13.4 and +26.6 kcal·mol^–1^. In this case, the most reactive Ga–O pairs are V–O3 and III–O2, with similar energy barriers and reaction energies (cf. Table 2): Δ*E*^⧧^ values equal to 41.7 and 41.9 kcal·mol^–1^ and Δ*E* values equal to +13.4 and +15.6 kcal·mol^–1^, respectively. It is interesting to note that, once the C–H activation has taken place on the V–O2 Ga–O pair, β-H transfer is more energy-demanding (Δ*E*^⧧^ = 49.0 kcal·mol^–1^ and Δ*E*= 26.6 kcal·mol^–1^) than when the C–H activation of propane takes place on the V–O3 Ga–O pair (Δ*E*^⧧^ = 41.7 kcal·mol^–1^ and Δ*E* = 13.4 kcal·mol^–1^). The other possible Ga–O pairs are I–O3 and II–O3; they are associated with rather high energy barriers of 49.1 and 51.8 kcal·mol^–1^ with reaction energies of 20.4 and 23.1 kcal·mol^–1^, respectively. The correlation between the energy barriers and reaction energies for the β-H transfer step for all of the evaluated sites is similar to that for the C–H activation previous reaction step (*R*^2^ = 0.76).

**Table 2:** Energy Barrier Heights (Δ*E*^⧧^) and Reaction Energies (Δ*E*) for the β-H Elimination Step (in kcal·mol^–1^) on the Evaluated Ga–O Pairs

Ga–O pair	Δ*E*^⧧^ (kcal·mol^–1^)	Δ*E* (kcal·mol^–1^)
I–O3	49.1	20.4
II–O3	51.8	23.1
III–O2	41.9	15.6
V–O2	49.0	26.6
V–O3	41.7	13.4

We also evaluated the energy for the decoordination of propene in all of the evaluated Ga sites. In all cases, it is an endoenergetic step by 10.2, 7.0, 15.5, 14.4, and 18.0 kcal·mol^–1^ on the I–O3, II–O3, III–O2, V–O2, and V–O3 Ga–O pairs. It is worth mentioning that this step is exoergic in Gibbs free energy (vide infra).

**Figure 2:**
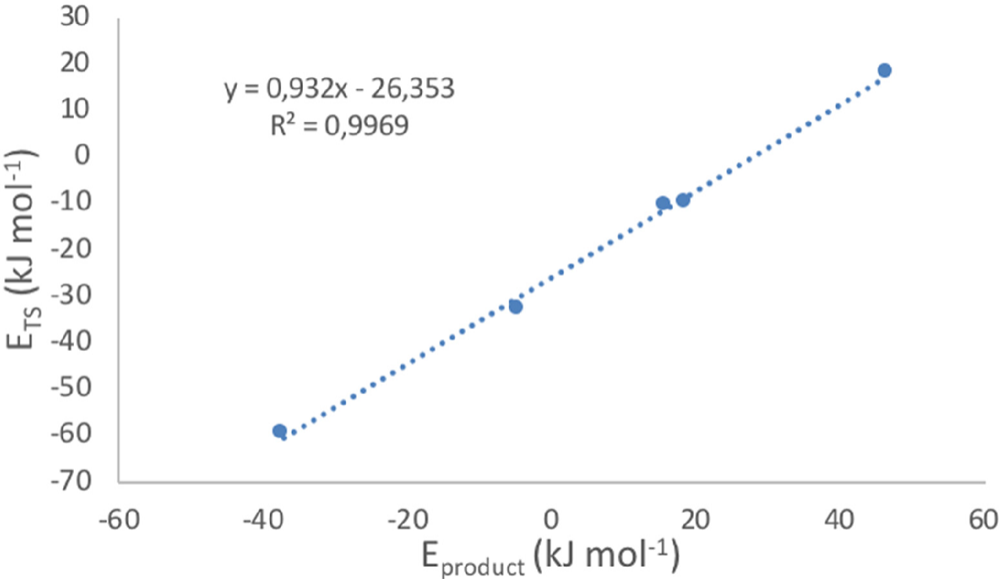
TS scaling for the β-H transfer step. *E*_TS_ versus *E*_product_ (both in kJ·mol^–1^) of the β-H transfer step with respect to the energy of the propane molecule and the respective initial Ga sites.

For this reaction step, however, we found that the TS scaling relationship holds very well (*R*^2^ = 0.997; Figure 2). This relationship relates the energy of a given TS and its product, in which the energies of both structures are referenced with respect to the initial reactant molecule and catalyst,^42,43^ in our case propane and the respective initial Ga sites.

### 2.5 H–H Coupling and H_2_ Formation

The last step corresponds to coupling of the hydride and the proton bonded to Ga and O, respectively, which regenerates the initial catalytic site along with H_2_. In this case, the most reactive Ga–O pair is II–O3, with the energy barrier for H–H coupling being equal to 27.3 kcal·mol^–1^, in an endoenergetic step of 7.7 kcal·mol^–1^. Three Ga–O pairs, namely, sites I–O3, III–O2, and V–O3, have similar reactivity. The reaction energies are endoenergetic and within 31.8–33.2 kcal·mol^–1^, while the energy barriers for H–H coupling on these three sites are within 43.1–48.9 kcal·mol^–1^. Finally, the Ga–O V–O2 pair, which was the most reactive site for the C–H activation of propane, has a high energy barrier for H–H coupling (73.8 kcal·mol^–1^) in a reaction step endothermic by 64.6 kcal·mol^–1^.

In order to obtain the BEP relationship for this reaction step, the opposite reaction to the H–H coupling step, i.e., H_2_ cleavage, needs to be evaluated. In this case, the BEP relationship between Δ*E*^⧧^ and Δ*E* of H_2_ cleavage is only followed to a certain extent (*R*^2^ = 0.69). Nevertheless, in this case, there is a very good correlation between Δ*E*^⧧^ and Δ*E* among for the forward reaction; however, it is difficult to interpret the physical meaning behind this correlation (Table 3).

**Table 3:** Energy Barrier Heights (Δ*E*^⧧^) and Reaction Energies (Δ*E*) for the H–H Coupling Reaction (in kcal·mol^–1^) on the Evaluated Ga–O Pairs

Ga–O pair	Δ*E*^⧧^ (kcal·mol^–1^)	Δ*E* (kcal·mol^–1^)
I–O3	48.9	33.1
II–O3	27.3	7.7
III–O2	44.4	31.8
V–O2	73.8	64.6
V–O3	43.1	33.2

### 2.6 Overall Catalytic Cycles for PDH on the Selected Sites

**Figure 3:**
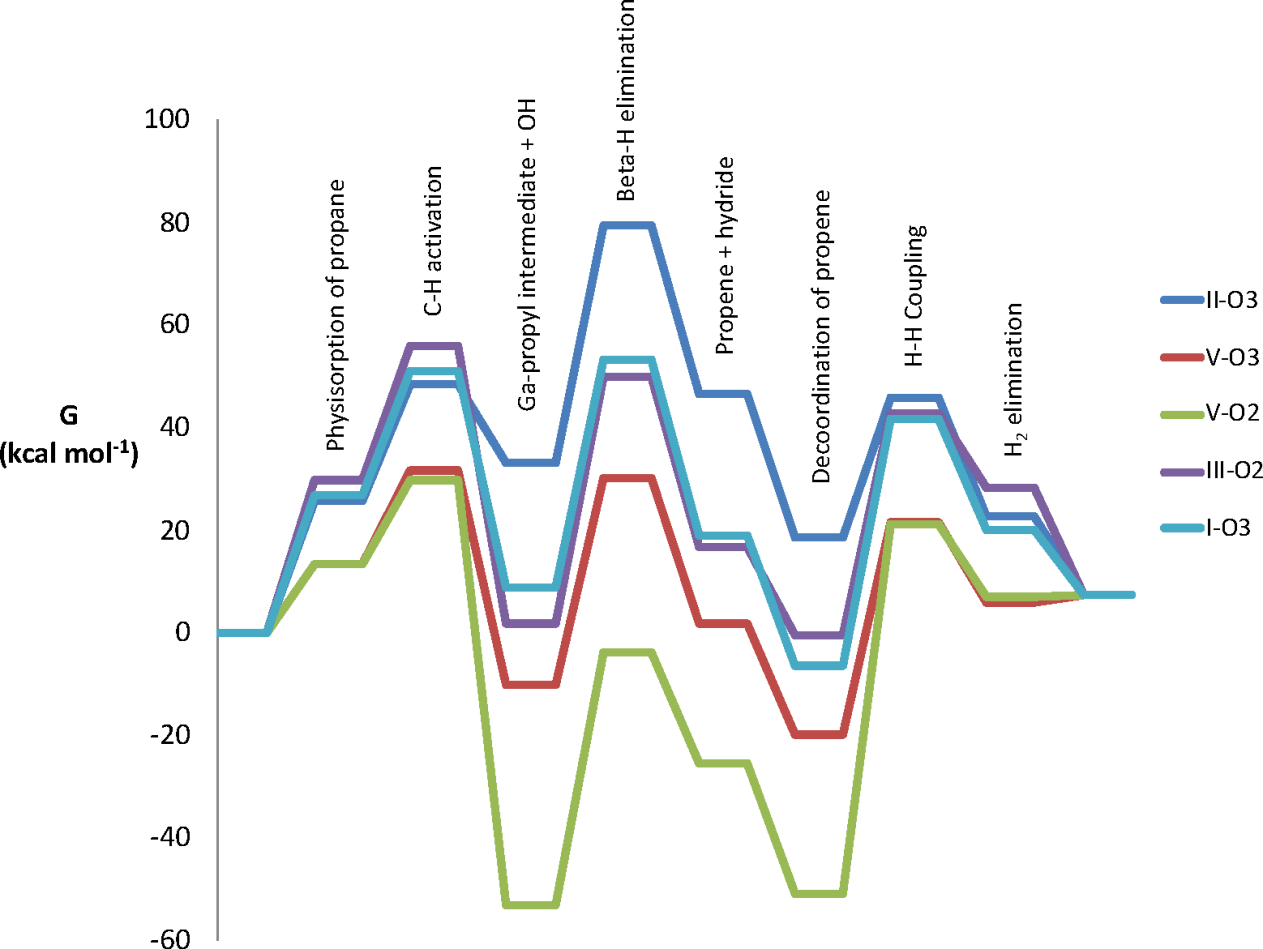
Gibbs energy profile of the PDH reaction on the five evaluated Ga–O pairs of sites (I–O3, II–O3, III–O2, V–O2, and V–O3). The reference Gibbs energy for the energy profile is the sum of the respective energies of the site and the energy of the propane molecule in the gas phase.

Finally, we can evaluate the overall reactivity in the dehydrogenation of propane for all of the evaluated sites, considering the three reaction steps previously described. The Gibbs energy profiles for all of the Ga–O pairs (I–O3, II–O3, III–O2, V–O2, and V–O3) are shown in Figure 3. The graph shows indeed a significant variability among the five evaluated sites. On the basis of the obtained Gibbs energy profile, we can compare the reactivity between the different sites.

Overall, the calculated reaction free energy is endergonic at 550 °C and 1 bar by 7.4 kcal·mol^–1^, in good agreement with the thermodynamics limitations of the PDH reaction because at this temperature the equilibrium conversion for propane is still of ca. 30% at 550 °C and 1 bar.^44^ In order to compare the catalytic activity of the different sites, we have used the energetic span model.^45^ In this model, the turnover frequency (TOF) of a catalytic cycle is a function of the energetic span (∂*E*), which depends on the energy of the TOF-determining transition state (TDTS), which in a simplified view is the TS with the highest energy in the Gibbs energy profile, and the TOF-determining intermediate (TDI), which is generally the most stable intermediate in the energy profile. Whenever the TDTS appears after the TDI, ∂*E* is the energy difference between these two steps, whereas when it is the reverse, Δ*G* of the reaction (Δ*G*_r_) is added to this difference, where the energetic span model (∂*E*) follows the equation
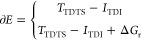
On the basis of the energetic span, we can then calculate the TOF of the reaction of interest by using the expression
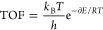
The former equation holds for exergonic reactions, leading in this case to a positive TOF value. Within this model, the TOF is understood as the catalytic flux, in analogy with Ohm’s law in electric circuits.^45^ A positive TOF is found for exergonic reactions, meaning that the catalytic flux goes forward, whereas for endergonic reactions, the TOF is negative because the catalytic flux goes backward.

Nevertheless, experimentally the TOF is defined differently. Because it is based on the conversion to products, it will always be a positive quantity. Indeed, for the Ga(III)/SiO_2_ catalyst, the reported initial experimental TOF is equal to 20.4 mol of propene per mole of Ga per hour under a kinetic regime (ca. conversion of 10%), despite the reaction being endergonic experimentally at 550 °C and 1 bar. Thus, in order to compare the catalytic activity for the evaluated sites to the experimental data in a semiquantitative way, we will make use the above-mentioned equation even though the Δ*G*_r_ term is positive in our case. For a full discussion of how we apply the TOF model for the current case, we refer the reader to the Supporting Information. We also refer to the work of Shaik and Kozuch, who developed the energetic span model, in which the meaning of the TOF within the model is discussed in depth.^45^

**Figure 4:**
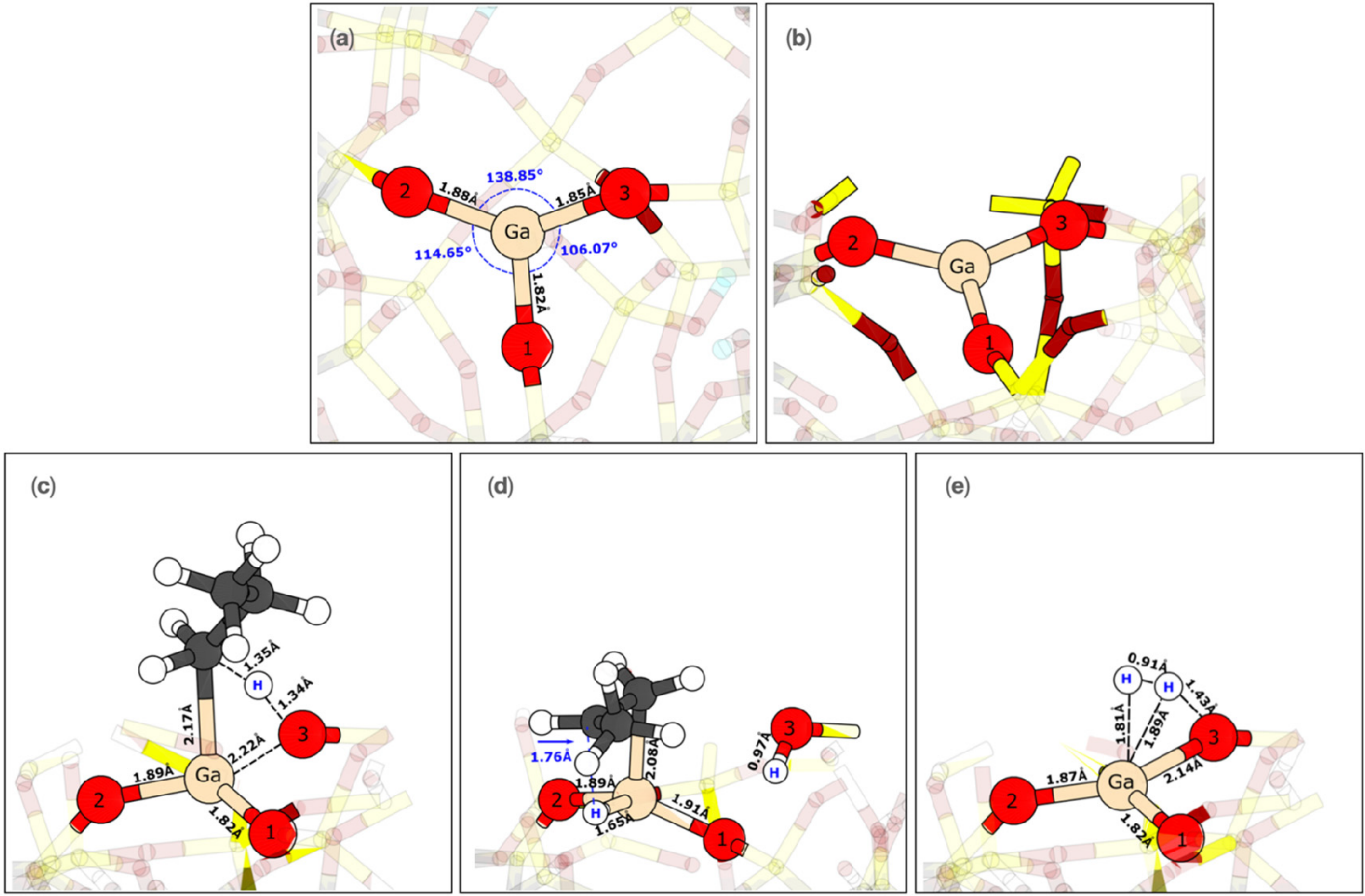
Top (a) and side (b) views of the Ga V site, with the corresponding labeling of the O sites. TS corresponding to the C–H activation of propane (c), β-H transfer (d), and H–H coupling of the V−O3 Ga−O pair (e) and their key geometrical features (in Å).

In any case, when using the rigorous application of the energetic span model, the trend of the reactivity found between the different sites stays the same as the one described here. For the Ga–O pair II–O3, the highest TS (TDS) in the energy profile corresponds to the β-H transfer step; it is located 79.3 kcal·mol^–1^ above the initial reactants, which are the most stable species of the catalytic cycle. Thus, in this case, the energetic span is equal to 79.3 kcal·mol^–1^ and the calculated TOF would be equal to 4.57 × 10^–5^ h^–1^. Therefore, this Ga–O pair would be inactive. Another Ga–O pair site that is unreactive is the V–O2 Ga–O pair but for a different reason. In this case, the initial C–H activation of propane is the TDTS, being located at 29.6 kcal·mol^–1^ with respect to the initial reactants, in a significantly exoergic step due to the significant release of strain, with the corresponding product being located at −53.1 kcal·mol^–1^ with respect to the same reference, with the latter species being the TDI of the catalytic cycle. Overall, considering the energy of the TDTS and TDI and the reaction energy, because in this case the TDI appears after the TDTS, the energetic span is equal to 90.1 kcal·mol^–1^ for the Ga–O pair V–O2. Thus, this site is also inactive, with a calculated TOF equal to 6.05 × 10^–8^ h^–1^. The III–O2 and I–O3 Ga–O pairs present rather similar Gibbs energy profiles. They present similar midrange relative energy barriers for the C–H activation, β-H transfer, and H–H coupling steps: 56.0 versus 48.6 kcal·mol^–1^, 48.2 versus 44.5 kcal·mol^–1^, and 43.3 versus 48.1 kcal·mol^–1^. For these two sites, the calculated energetic span is equal to 63.9 and 67.2 kcal·mol^–1^, which would correspond to TOFs equal to 0.58 and 0.08 h^–1^, respectively; thus, both sites would be active in the PDH reaction. Finally, the most active Ga–O pair among all of the evaluated sites would be the V–O3 one. This site presents a rather feasible C–H activation step at 823.15 K, with a relative low energy barrier equal to 31.7 kcal·mol^–1^. The corresponding TS is the TDTS of the catalytic cycle. This C–H activation relative energy barrier value is similar to the one that we found for the V–O2 Ga–O pair (29.6 kcal·mol^–1^). Nevertheless, in this case, the product of the C–H activation is exergonic but to a significantly less extent than that for the V–O2 pair: −10.3 versus −53.1 kcal·mol^–1^. The subsequent β-H transfer and H–H coupling also present affordable relative energy barriers at 550 °C: 40.3 and 41.3 kcal·mol^–1^, respectively. In this case, the TDI appears after the TDTS; it corresponds to the gallium hydride species, with a relative energy equal to −19.8 kcal·mol^–1^. In this case, the energetic span is equal to 58.9 kcal·mol^–1^, corresponding to a TOF equal to 12.5 h^–1^, which is very similar to the TOF obtained experimentally for the PDH reaction: 20.4 h^–1^.^16^ Despite all of the approximations and considerations made to calculate the energetic span and the resulting TOF, it is fair to conclude that V–O3 is the most active among all of the evaluated sites in PDH. A graphical representation of the Ga site V with the corresponding labeling of the O sites is given in Figure 4a,b, whereas the three TSs for the Ga V–O3 pair—C–H activation of propane, β-H transfer, and H–H coupling—and its key geometrical features are shown in parts c–e of Figure 4, respectively. Further optimized structures along the PDH pathway for the III–O2 and I–O3 sites are also depicted in Figure S8.

Geometrically, this V–O3 Ga–O pair has a Ga–O distance significantly elongated, being equal to 1.854 Å. Nevertheless, it is not elongated as the V–O2 pair, in which the distance is equal to 1.880 Å. Therefore, on the basis of the distance, the Ga–O pair is reactive but not too much. Concerning the two O–Ga–O angles in which the V–O3 Ga–O pair is involved, they take quite different values: being equal to 106.0 and 138.9°. Thus, the V–O3 Ga–O pair and the site as a whole is highly asymmetric because the remaining O–Ga–O angle of the Ga–V site is equal to 114.6°. In comparison to the other sites, as evidenced by the O–Ga–O angle sites: site V is the most asymmetric among all of them (see Figure S9 for a histogram of the Ga–O distances for all of the sites). Finally, the dihedral angles in which the Ga–O pair is involved in one of the ends are equal to 171.1 and 173.9°. The other dihedral angle takes a value equal to 171.6°. Thus, all of the dihedral angles of this site are close to 180°, meaning the site is highly coplanar. In comparison, the other sites show coplanarity similar to that of site II, and it is only slightly less coplanar than site I, which is the most coplanar of all of the sites (all of the dihedral angles are close to 180°). In contrast, sites III and III-mod are quite far from coplanarity, with allof the dihedral O–Ga–O–O angles taking values lower than 160°.

From geometrical analysis, one could argue that both the Ga–O distances and O–Ga–O angles could be used as a descriptor for the C–H activation as well as the H–H activation.

In addition, the results indicate that the V–O3 Ga–O pair represents a good model in order to describe the overall activity of the Ga(III)/SiO_2_ catalyst in the PDH reaction. It also has an intermediate strain, yielding the highest activity. The trend in the reactivity of the evaluated Ga–O pairs is the following: V–O3 > III–O2 > I–O3 > II–O3 > V–O2. Overall, for the C–H activation of propane, the sites that are more strained and more favorable to be cleaved had low energy barriers and significantly more favored reaction energies, i.e., significantly exothermic. Conversely, for the H–H coupling step, the Ga–O pair is formed again and thus the sites that were more favorable for the C–H activation of propane now become less favorable for this step. In addition, if the initial C–H activation is too exothermic, this leads to very stable intermediates in the Gibbs energy profile, which decreases the overall catalytic activity of that specific Ga–O pair.

## 3 Conclusions

Isolated Ga(III) sites dispersed on silica are rather active and selective catalysts for the PDH reaction. After construction of the Ga(III) sites on SiO_2_ amorphous periodic models, we have evaluated the reactivity of a variety of Ga–O pairs with different degrees of strain. For the selected sites, we evaluated three reaction steps, namely, the C–H activation of propane, β-H transfer step, and H–H coupling. We considered tri- and tetracoordinated Ga with one additional siloxane group coordinated to the Ga center because these are the proposed initial catalytic sites in the silica-supported well-defined Ga(III) PDH catalyst. For the tetracoordinated sites, the additional siloxane group coordinated to Ga does not seem to play a key role in the PDH reaction on the evaluated catalytic system. After the C–H activation step of propane, the Ga···O interaction between the Ga center and the O site of the siloxane group is lost, and its effect on the energetics is rather small. For the three evaluated reaction steps, we have found that the BEP relationship holds to certain extent for the C–H and H–H activation steps. In addition, the TS scaling holds very well for the β-H transfer step. This is rather interesting because, if true for other single sites based on elements other than Ga, it would allow screening of the reactivity of the different sites only via evaluation of the thermodynamics of the three proposed reaction steps in the PDH reaction. Thus, our current results can serve as a basis for the future computational screening of PDH silica-supported single-site catalysts, especially for those centers in which the β-H transfer is rather energy-demanding. Concerning the overall catalytic activity of the evaluated sites using the energetic span model, we have found that the strain reduces significantly the C–H activation of propane. Nevertheless, if the strain is too high and the product of the C–H activation of propane is too stable, that compromises the overall catalytic activity in the dehydrogenation of propane because the subsequent β-H transfer and H–H coupling reaction steps, as well as the C–H activation of propane, become significantly more energy-demanding, increasing the energetic span and significantly decreasing the activity of the evaluated Ga–O pair. Thus, a compromise is needed between the strain, meaning an elongated Ga–O pair for the effective cleavage of the C–H bond of propane, but not too much in order to regenerate the reactive site effectively. Among all of the evaluated Ga(III)/SiO_2_ sites, the one displaying the highest catalytic activity is Ga–O V–O3, which has a rather elongated Ga–O bond, and it is embedded in a highly asymmetric Ga(III) site close to coplanarity, as evidenced by the difference in the O–Ga–O bonds and the O–Ga–O–O dihedral angles close to 180°.

## 4 Computational Methods

Density functional theory calculations based on the Gaussian and plane-wave (GPW) formalism^46^ were carried out using the *Quickstep* (QS) module^47^ of the *CP2K* program package.^48,49^ The functional chosen was Perdew–Burke–Ernzerhof (PBE)^50−52^ with short-range Gaussian double-ζ basis sets^53^ optimized from molecular calculations. The energy cutoff of the auxiliary plane-wave basis set was set to 500 Ry. The Goedecker–Teter–Hutter pseudopotentials^54−56^ were used. The orbital transformation method was applied.^57,58^ A tetragonal simulation box of base area 21.4 Å × 21.4 Å and thickness 34.2 Å (ca. 24 Å of which corresponds to a vacuum slab added in order to avoid interactions between images in the *z* direction) was used.^36^ Ground-state structures were obtained by energy minimization with the BFGS algorithm.^59−63^ The initial TS guesses were generally obtained from CI-NEB^64−68^ band calculations. TS structure optimizations were performed using the dimer method^69,70^ with the conjugate gradient optimizer and the two-point-based line search. In a few cases, in addition to the correct imaginary frequency along the reaction coordinate, minor imaginary components were obtained that could not be avoided. However, they are expected to have a minimal impact on the reported energies.

## Supporting Information Available

Geometrical characteristics of each Ga site, relative stability of the tricoordinated sites, BEP relationship for the C–H activation step of propane, additional representative optimized structures, histogram comparing the Ga–O distances among sites, and explanation of how the span model was applied in the current work (PDF)
