# Supplementary material for: Strain in Silica-Supported Ga(III) Sites: Neither Too Much nor Too Little for Propane Dehydrogenation Catalytic Activity
Source: Inorg Chem. 2021 Feb 5;60(10):6865–74. doi: 10.1021/acs.inorgchem.0c03135 (PMC8483445; doi:10.1021/acs.inorgchem.0c03135)
Supplement: Supplementary file 1 — ic0c03135_si_001.pdf [file ic0c03135_si_001.pdf]

Electronic Supplementary Information

Strain in Silica-Supported Ga (III) Sites: neither too much nor too little for Propane Dehydrogenation Catalytic Activity

C. S. Praveen,[1,2,3] A. P. Borosy,[1] C. Copéret[1] and A. Comas-Vives[4*]

[1]Department of Chemistry and Applied Biosciences, ETH Zürich, Vladimir Prelog-Weg 1-5, CH-8093 Zürich, Switzerland

Present addresses: [2]International School of Photonics, Cochin University of Science and Technology, University Road, South Kalamassery, Kalamassery, Ernakulam, Kerala 682022, India. [3]Inter University Centre for Nano Materials and Devices, Cochin University of Science and Technology, University Road, South Kalamassery, Kalamassery, Ernakulam, Kerala 682022, India.

[4]Department of Chemistry, Universitat Autònoma de Barcelona, 08193 Cerdanyola del Vallès, Catalonia, Spain

Ga – Site – I

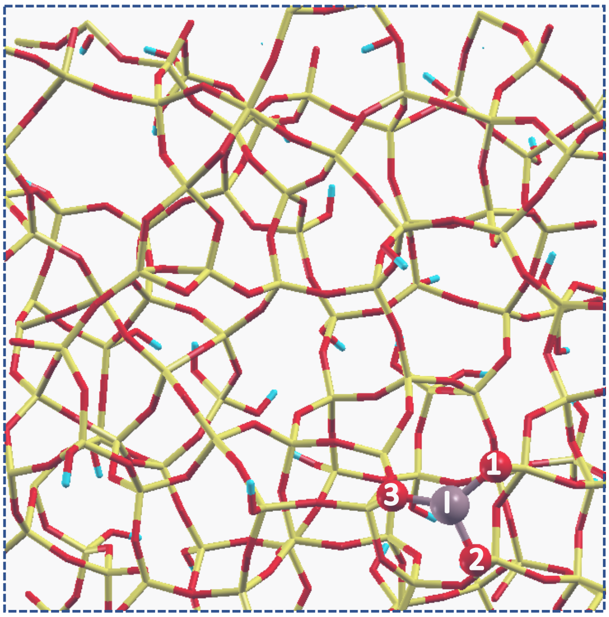

Label	Distance	Angle	Dihedral
Ga-O1	1.858		
Ga-O2	1.851		
Ga-O3	1.817		
O3-Ga-O2		123.5	
O3-Ga-O1		131.64	
O2-Ga-O1		104.86	
O1-Ga-O2-O3			179.42
O2-Ga-O1-O3			179.36
O1-Ga-O3-O2			179.25

Figure S1: Optimized structure and structural analysis of Ga-Site-I. All distances are in Angstrom and the angles are in degree. Atoms are color codes as follows- Silicon (Yellow), Oxygen (Red), Hydrogen (Blue), and Gallium (Grey). Only the Ga (III), O1, O2 and O3 are shown as solid balls for ease of reference.

Ga – Site – II

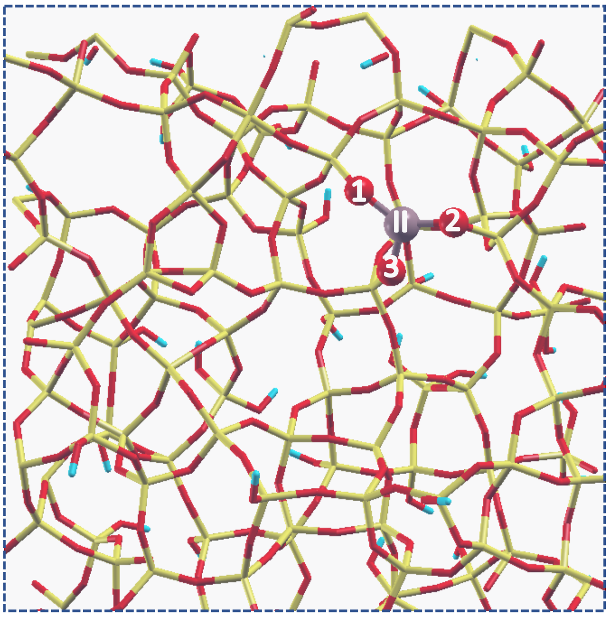

Label	Distance	Angle	Dihedral
Ga-O1	1.831		
Ga-O2	1.808		
Ga-O3	1.848		
O1-Ga-O3		114.54	
O1-Ga-O2		132.81	
O2-Ga-O3		112.16	
O1-Ga-O2-O3			171.39
O1-Ga-O3-O2			173.07
O2-Ga-O1-O3			171.23

Figure S2: Optimized structure and structural analysis of Ga-Site-II.

Ga – Site – III

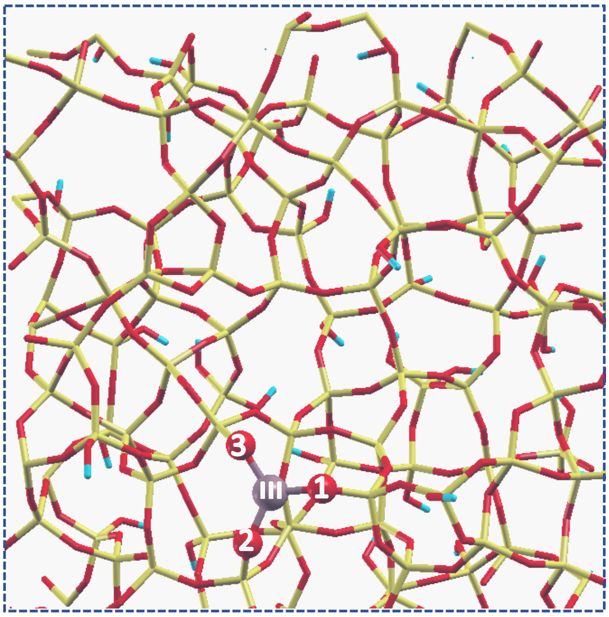

Label	Distance	Angle	Dihedral
Ga-O1	1.844		
Ga-O2	1.856		
Ga-O3	1.862		
O3-Ga-O2		123.734	
O3-Ga-O1		114.783	
O2-Ga-O1		117.622	
O1-Ga-O2-O3			156.683
O2-Ga-O1-O3			158.743
O1-Ga-O3-O2			157.277

Figure S3: Optimized structure and structural analysis of Ga-Site-III.

Ga – Site – III-m

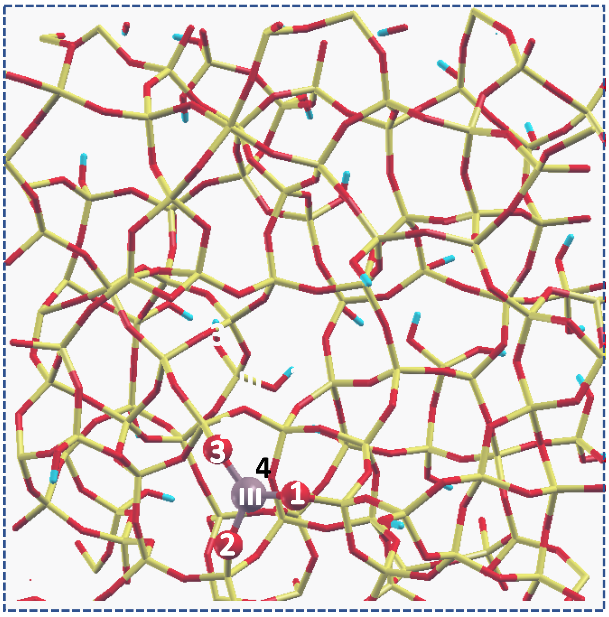

Label	Distance	Angle	Dihedral
Ga-O1	1.851		
Ga-O2	1.868		
Ga-O3	1.864		
Ga-O4	2.479		
O3-Ga-O2		123.503	
O3-Ga-O1		117.267	
O2-Ga-O1		113.285	
O4-Ga-O3		92.650	
O4-Ga-O2		99.078	
O4-Ga-O1		103.184	
O1-Ga-O2-O3			152.006
O2-Ga-O1-O3			153.876
O1-Ga-O3-O2			150.985
O4-Ga-O3-O2			102.714
O4-Ga-O2-O3			99.318
O4-Ga-O3-O1			106.303
O4-Ga-O1-O3			100.030
O4-Ga-O2-O1			108.675
O4-Ga-O1-O2			106.095

Figure S4: Optimized structure and structural analysis of Ga-Site-III-m.

Ga – Site – V

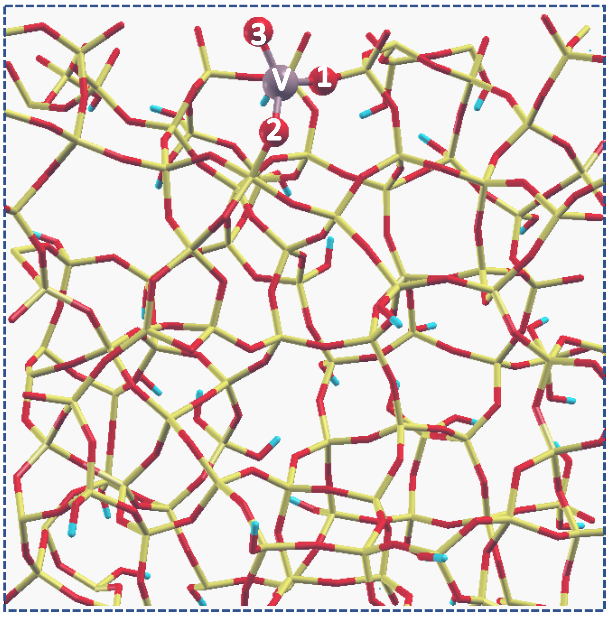

Label	Distance	Angle	Dihedral
Ga-O1	1.816		
Ga-O2	1.880		
Ga-O3	1.854		
O3-Ga-O2		138.854	
O3-Ga-O1		106.0721	
O2-Ga-O1		114.645	
O1-Ga-O2-O3			171.107
O2-Ga-O1-O3			173.924
O1-Ga-O3-O2			171.592

Figure S5: Optimized structure and structural analysis of Ga-Site-V.

Ga – Site – III with SiH_3

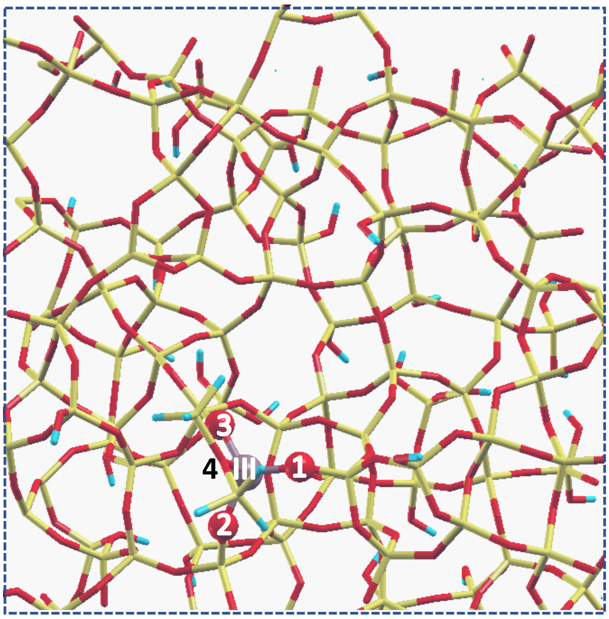

Label	Distance	Angle	Dihedral
Ga-O1	1.845		
Ga-O2	1.883		
Ga-O3	1.889		
Ga-O4	2.036		
O1-Ga-O2		126.121	
O1-Ga-O3		114.681	
O2-Ga-O3		118.064	
O1-Ga-O2-O3			167.130
O1-Ga-O3-O2			168.579
O2-Ga-O1-O3			167.507

Figure S6: Optimized structure and structural analysis of Ga – Site – III with SiH_3
.

Site	**Relative Energy (kcal mol^{-1})**
Site I	16.9
Site II	14.5
Site III	**0.0**
Site III-mod	2.4
Site V	24.6

Table S1. The relative stability of the initial tri-coordinate sites - since they are the only structures- which allows a direct comparison regarding stability

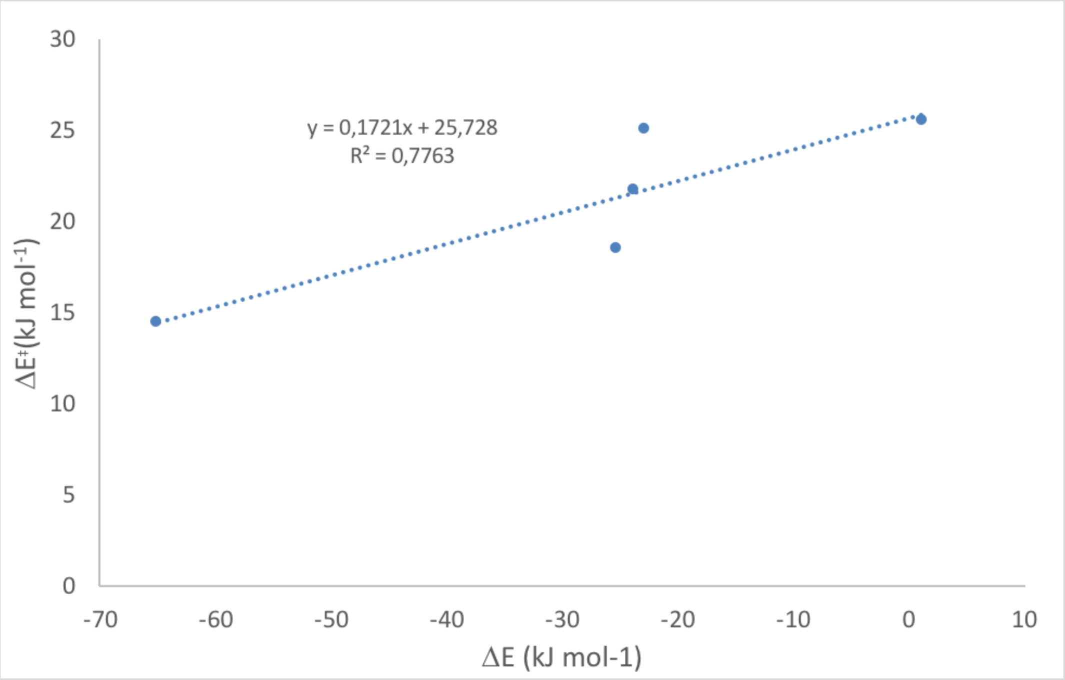

Figure S7. Brønsted-Evans-Polanyi relationship for the C-H activation of propane on the different evaluated Ga-O pairs.

It is to be noted that, the mod-III-O2 deviates from the correlation. This is because, even though it has the same transition-state and final products than the III-O2 site the initial state is different. In the mod-III-O2 site an additional siloxane coordinates to the Ga center in comparison to the III-O2 site. This modifies the energy of the initial reactant to build the BEP relationship, but not the final product and the transition-state since this siloxane interaction is lost in both structures. Since the BEP relationship for the mod-III-O2 has this additional siloxane interaction in the initial structure, the mod-III-O2 site deviates from the correlation.

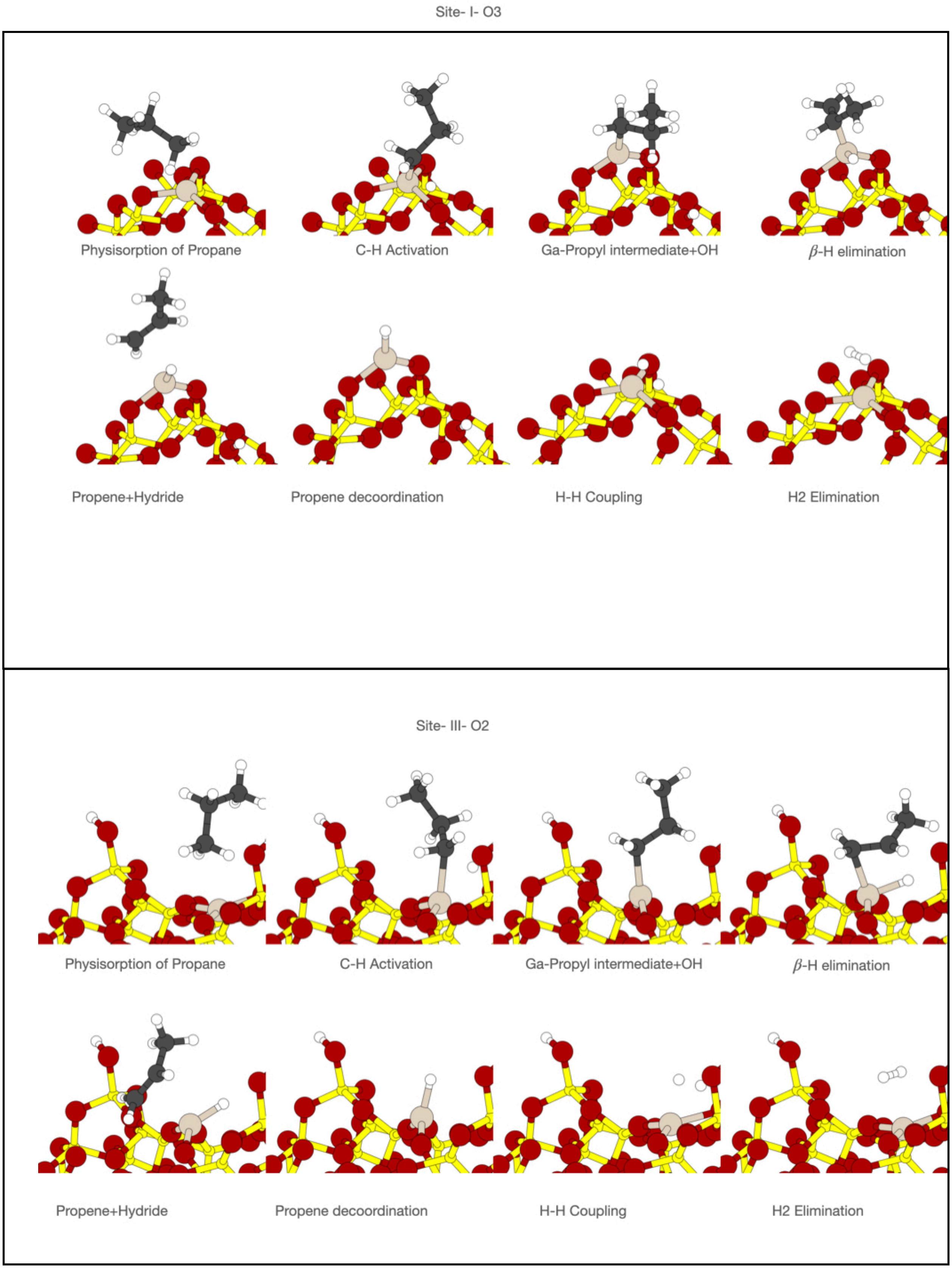

Figure S8. Representative optimized structures corresponding to the reaction energy profile of Site (I)-O3 and Site (III)-O2 – See Figure 3 in text for the reaction profile.

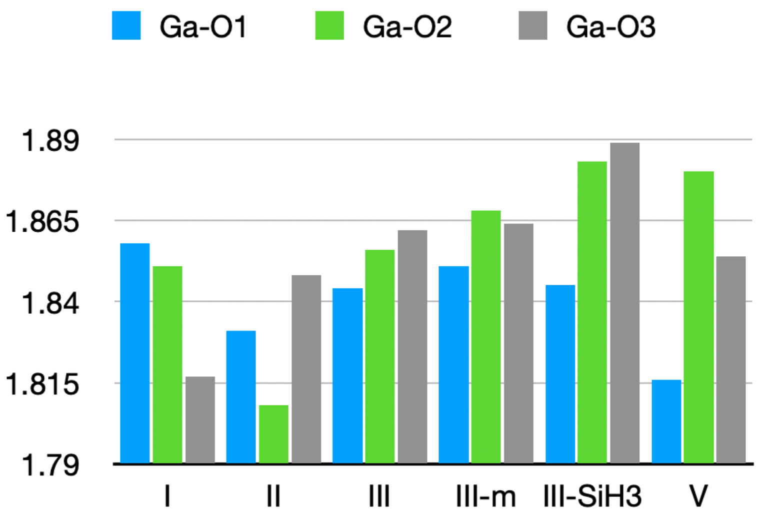

Figure S9. Histogram demonstrating the Ga-O distances for all the sites studied.

Application of the energetic span model

The TOF, according to the IUPAC Gold book, is defined as "Commonly called the turnover number, N, and defined, as in enzyme catalysis, as molecules reacting per active site in unit time". Thus, indeed from this definition, it should be a positive quantity. Nevertheless, within the span model, the TOF, as discussed by Kozuch and Shaik by analogy to Ohm's law, the TOF can be seen as a catalytic-flux law.[1]

$$TOF = \frac{k_B T}{h} \frac{e^{-\frac{\Delta G_r}{RT}} - 1}{\sum_{i,j=1}^{N} e^{\frac{(T_i - I_j - \delta G'_{ij})}{RT}}}$$

With:

$$\delta G'_{ij} = \begin{cases} \Delta G_r \ if \ i > j \\ 0 \ if \ i \leq j \end{cases}$$

According to this definition, if the $\Delta G_r > 0$ (endergonic reaction), then the exponential term exp(-$\Delta G_r/RT$) is smaller than -1 and therefore the numerator is negative and the overall TOF is also negative. Thus, in this case, the catalytic flux, gives a negative TOF, which means the current flow backwards. Conversely, for exergonic reactions using the equation above, the TOF is positive meaning the catalytic flux goes forward. However, the equation above is usually simplified if the reaction is exothermic, since in this case the exp(-$\Delta G_r/RT$) >> -1. Therefore, the -1 term can be neglected leading to the following equation:

$$TOF = \frac{k_B T}{h} e^{-\frac{\partial E}{RT}}$$

In which:

$$\partial E = \begin{cases} T_{TDS} - I_{TDS} \\ 0 \ if \ \ i \leq j \end{cases}$$

In our case, we have an endergonic reaction. Our calculated thermodynamics (based on ab initio calculations) for the PDH reaction calculated is endergonic by 7.4 kcal.mol^{-1}, in agreement with experiment that shows that the conversion is still at 30 % at 550 °C and 1 bar of pressure. Therefore, and rigorously speaking the simplification cannot be made. Nevertheless, we would like to get a positive TOF comparable to experiment, which uses the IUPAC definition, and also compare the catalytic activity of the evaluated sites. Therefore, we used the simplified form of the equation, in which d*E* can be seen as the apparent Gibbs activation energy of the catalytic cycle. This leads to a positive TOF and compares well with experimental data, i.e. same order of magnitude, although the result must be taken with care due to the simplification we did in order to obtain a positive TOF base on the computational calculations.

Reference

1. Kozuch, S.; Shaik, S., How to Conceptualize Catalytic Cycles? The Energetic Span Model. *Acc. Chem. Res.* **2011,** *44* (2), 101-110.
